# Supplementary material for: Antagonistic Effects of Actin-Specific Toxins on Salmonella Typhimurium Invasion into Mammalian Cells
Source: Biomolecules. 2024 Nov 9;14(11):1428. doi: 10.3390/biom14111428 (PMC11591686; doi:10.3390/biom14111428)
Supplement: Supplementary file 1 [file biomolecules-14-01428-s001.zip › Video Legends.pdf]

Video Legends

Video S1. Directional movement of VopF reflecting on its pointed-end actin elongation activity is not impaired by the inactive ACD. A multi-stack montage of time-lapse images of a peripheral region of an individual XTC cell expressing low levels of EGFP-VopF and treated with PA and inactive LF_N-ACD EE1990,1992AA mutant (at 2.5 and 1 nM final concentrations, respectively). Images were collected using TIRF microscopy at 0.5 s intervals for 2 min for each 15-min time point (only the first 1.5 min is shown for each) with 13 min intervals between the adjacent time points. Scale bars are 5 μm.

Video S2. Active ACD inhibits VopF pointed-end actin elongation activity. A multi-stack montage of time-lapse images of a peripheral region of an individual XTC cell expressing low levels of EGFP-VopF and treated with PA and active LF_N-ACD (at 2.5 and 1 nM final concentrations, respectively). Images were collected using TIRF microscopy at 0.5 s intervals for 2 min for each 15-min time point (only the first 1.5 min is shown for each) with 13 min intervals between the adjacent time points. Scale bars are 5 μm.
